# Meta-Regression Analyses, Meta-Analyses, and Trial Sequential Analyses of the Effects of Supplementation with Beta-Carotene, Vitamin A, and Vitamin E Singly or in Different Combinations on All-Cause Mortality: Do We Have Evidence for Lack of Harm?

**DOI:** 10.1371/journal.pone.0074558

**Published:** 2013-09-06

**Authors:** Goran Bjelakovic, Dimitrinka Nikolova, Christian Gluud

**Affiliations:** 1 The Cochrane Hepato-Biliary Group, Copenhagen Trial Unit, Centre for Clinical Intervention Research, Rigshospitalet, Copenhagen University Hospital, Copenhagen, Denmark; 2 Department of Internal Medicine, Gastroenterology and Hepatology, Medical Faculty, University of Nis, Niš, Serbia; 3 The Copenhagen Trial Unit, Centre for Clinical Intervention Research, Rigshospitalet, Copenhagen University Hospital, Copenhagen, Denmark; CUNY, United States of America

## Abstract

**Background and Aims:**

Evidence shows that antioxidant supplements may increase mortality. Our aims were to assess whether different doses of beta-carotene, vitamin A, and vitamin E affect mortality in primary and secondary prevention randomized clinical trials with low risk of bias.

**Methods:**

The present study is based on our 2012 Cochrane systematic review analyzing beneficial and harmful effects of antioxidant supplements in adults. Using random-effects meta-analyses, meta-regression analyses, and trial sequential analyses, we examined the association between beta-carotene, vitamin A, and vitamin E, and mortality according to their daily doses and doses below and above the recommended daily allowances (RDA).

**Results:**

We included 53 randomized trials with low risk of bias (241,883 participants, aged 18 to 103 years, 44.6% women) assessing beta-carotene, vitamin A, and vitamin E. Meta-regression analysis showed that the dose of vitamin A was significantly positively associated with all-cause mortality. Beta-carotene in a dose above 9.6 mg significantly increased mortality (relative risk (RR) 1.06, 95% confidence interval (CI) 1.02 to 1.09, I^2^ = 13%). Vitamin A in a dose above the RDA (> 800 µg) did not significantly influence mortality (RR 1.08, 95% CI 0.98 to 1.19, I^2^ = 53%). Vitamin E in a dose above the RDA (> 15 mg) significantly increased mortality (RR 1.03, 95% CI 1.00 to 1.05, I^2^ = 0%). Doses below the RDAs did not affect mortality, but data were sparse.

**Conclusions:**

Beta-carotene and vitamin E in doses higher than the RDA seem to significantly increase mortality, whereas we lack information on vitamin A. Dose of vitamin A was significantly associated with increased mortality in meta-regression. We lack information on doses below the RDA.

**Background:**

All essential compounds to stay healthy cannot be synthesized in our body. Therefore, these compounds must be taken through our diet or obtained in other ways [1]. Oxidative stress has been suggested to cause a variety of diseases [2]. Therefore, it is speculated that antioxidant supplements could have a potential role in preventing diseases and death. Despite the fact that a normal diet in high-income countries may provide sufficient amounts of antioxidants [3,4], more than one third of adults regularly take antioxidant supplements [5,6].

## Introduction

The preventive potential of antioxidants has been studied in a large number of trials [7]. The results so far have remained largely inconclusive [7]. Systematic reviews of randomized clinical trials have shown that some antioxidant supplements may increase mortality [7,8].

The present study is based on the results of our recently updated Cochrane systematic review of primary and secondary prevention randomized clinical trials with low risk of bias showing that all-cause mortality was significantly increased by the administration of beta-carotene, vitamin E, and possibly vitamin A [8]. The review results urged us to assess whether doses of beta-carotene, vitamin E, and vitamin A influenced all-cause mortality, using meta-regression analyses. We also compared the influence of daily dose within the recommended dietary (daily) allowance (RDA) (low dose) to daily dose above the RDA (high dose) of beta-carotene, or vitamin A, or vitamin E on all-cause mortality in meta-analyses. To control the risks of type I and type II errors in meta-analyses, we used trial sequential analyses to assess whether the level of evidence was sufficient to make conclusions (i.e., whether the required information size was reached or if any of the trial sequential monitoring boundaries were crossed before the required information size was reached) [9–13].

## Methods

Review methods are described in detail in two earlier publications [7,8]. Briefly, we identified relevant randomized clinical trials by searching The Cochrane Library, MEDLINE, Embase, LILACS, Science Citation Index Expanded, and Conference Proceedings Citation Index-Science from inception to February 2011 [8]. We scanned bibliographies of relevant publications and asked experts and pharmaceutical companies for additional trials. We considered for inclusion primary and secondary prevention randomized clinical trials in adults (aged ≥ 18 years) comparing beta-carotene, vitamin A, vitamin C, vitamin E, and selenium at any dose, duration, and route of administration versus placebo. The antioxidants could have been administered separately or in any combination, or in combination with other vitamins or trace elements without antioxidant function. Concomitant interventions were allowed when used equally in all the intervention groups of the trial.

For the present study we selected only the randomized primary or secondary prevention trials with low risk of bias where beta-carotene, vitamin A, and vitamin E were compared with placebo. This was in order to assess trials with the lowest risk of bias, that is overestimation of benefits and underestimation of harms (see below) [8]. Trials including general and healthy populations were defined as primary prevention [14]. Trials in which investigators mentioned a specific disease as inclusion criterion for the participants were defined as secondary prevention [14]. We excluded tertiary prevention (treatment) trials, like trials on acute, infectious, or malignant diseases, except for non-melanoma skin cancer [8]. Our only outcome was all-cause mortality at maximum follow-up. Two review authors independently assessed trial eligibility without blinding of the study authors. We listed excluded trials with the reasons for exclusion. Disagreement was resolved by discussion or in consultation with a third author. We contacted authors of the trials for missing information.

Due to the risk of overestimation of beneficial intervention effects and underestimation of harms in randomized clinical trials with unclear or inadequate methodological quality, our present analyses only included trials with low risk of bias [15–18]. The risk of bias was assessed in regard to generation of the allocation sequence, allocation concealment, blinding, incomplete outcome data, outcome reporting, and other bias like industry or academic bias [15–19]. The allocation sequence generation was classified as adequate if based on a table of random numbers or was computer-generated. The allocation concealment was classified as adequate if the allocation of participants involved a central independent unit, on-site locked computer, or identically appearing, numbered, drug bottles prepared by an independent investigator. Sealed envelopes were considered adequate if they were opaque and serially numbered. Blinding was classified as adequate if participants, personnel, and outcome assessors were blinded and the assessment of outcomes was not likely to be influenced by lack of blinding. The description of incomplete outcome data was classified as adequate if missing data were unlikely to make treatment effects depart from plausible values. Outcome reporting was classified as adequate if reported outcomes were pre-defined or clinically relevant and expected. We also considered whether the trial was free of other components that could put it at risk of bias. Trials with adequate assessments in all of the mentioned bias risks domains were considered as having low risk of bias.

### Doses of antioxidant supplements

We used the RDA provided by the Institute of Medicine of the National Academies [20,21] to separate trials of low experimental dose (≤ RDA) from trials of high experimental dose (> RDA). The RDA is the dietary intake level that is sufficient to meet the nutrient requirement of nearly all healthy individuals in a particular life stage and sex group [22]. The RDA is determined based upon the estimated average requirement, defined as a nutrient intake value that is estimated to meet the requirements of half the healthy individuals in a group plus twice the standard deviation [22].

RDAs for vitamin A and vitamin E are displayed in Table 2. No RDA has been established for beta-carotene [20,21]. The Institute of Medicine has proposed a retinol activity equivalent (RAE) of 12 mg of beta-carotene, equivalent to the activity of 1000 µg of all-trans retinol [22]. Therefore, we used the dose of 9.6 mg beta-carotene as the ‘RDA’ for beta-carotene equivalent to a dose of vitamin A of 800 µg. This RDA is close to the vitamin A RDA for men and women. We also recorded how many trials used doses above the tolerable upper intake level (TUIL), defined as the highest level of nutrient intake, likely to pose no risk of adverse health effects for almost all individuals in the general population [22,23].

### Statistical analyses

We conducted the systematic review according to the recommendations of The Cochrane Handbook for Systematic Reviews of Interventions [15]. For the present analyses, we used RevMan 5.2 (The Nordic Cochrane Centre, Copenhagen) [24], Trial Sequential Analysis version 0.9 beta (The Copenhagen Trial Unit, Copenhagen) [25], STATA 8.2 (STATA Corp, College Station, Tex), and Sigma Stat 3.0 (SPSS Inc, Chicago, Ill). We analyzed the data with a random-effects model meta-analysis calculating the relative risk (RR) with 95% confidence intervals (CI). Heterogeneity was explored using a chi-squared test, and the quantity of heterogeneity was measured using the I^2^ statistic [26]. Random-effects meta-regression analyses were performed to assess potential covariates that could predict intertrial heterogeneity, i.e., the covariates that were statistically associated with estimated intervention effects. The included covariates were daily dose of beta-carotene, daily dose of vitamin A, daily dose of vitamin E, type of prevention (primary or secondary), and the way of taking the supplements, i.e., singly or combined. We calculated weighted averages for duration of intervention and duration of the follow-up period.

### Trial sequential analyses (TSA)

A meta-analysis may result in type I errors and type II errors if data are sparse or if there is repeated testing for significance when new trials are added to it [9–13,27,28]. In a single trial, interim analyses increase the risk of type I errors. To avoid type I errors, group sequential monitoring boundaries [29] are constructed to decide whether a trial could be terminated early in the case of a sufficiently small P value, i.e., what we may see is a cumulative Z-curve crossing the sequential monitoring boundary either for benefit or harm. Trial sequential monitoring boundaries can, in a comparable way, be applied to meta-analysis [9–13]. Trial sequential monitoring boundaries are based on the calculation of a required information size and the formation of trial sequential monitoring boundaries for benefit, harm, and futility [9–13,28]. The methods for adjusting thresholds for significance (i.e., controlling the type I error) are built upon the ‘group sequential analysis’ methodology [10]. An interim look at the data of a meta-analysis is necessitated when data from new clinical trials are added. This happens at arbitrary intervals, and the number of added patients varies and cannot be predicted. Lan and DeMets extended the ‘group sequential’ methodology of repeated significance testing in a single randomized trial to ‘sequential analysis’ in order to allow for flexible, unplanned *interim analyses* [10]. This flexibility has been used for trial sequential analyses of meta-analyses, where trials are added in the sequence they are published (by year and alphabetically according to the family name of the first author if more trials are published in the same year). With the TSA program, trial sequential monitoring boundaries for benefits, harms, and futility are constructed [10]. If the cumulative Z-curve crosses any of the trial sequential monitoring boundaries for benefit, harm, or futility, sufficient evidence is reached and no further trials may be needed. If the cumulative Z-curve does not cross the trial sequential monitoring boundaries for benefit, or harm, or futility, there is insufficient evidence to reach any conclusion on the intervention effect. If the cumulative Z-curve does not reach the trial sequential monitoring boundary for futility, we have too sparse data to declare that the intervention does not cause benefit or harm [30].

We conducted trial sequential analyses to assess the risk of random errors due to sparse data and repetitive testing of cumulative meta-analyses [10,30]. The diversity (D^2^)-adjusted required information size (DARIS) and the adjacent trial sequential monitoring boundaries were calculated assuming a mortality of 10% in the placebo group [8]; an anticipated intervention effect of 5% relative risk reduction in the antioxidant supplemented group; a type I error of 5%; a type II error of 20% (80% power); and the diversity of the meta-analysis [28]. In the case when a trial sequential analysis was difficult to interpret because the distance between the accrued information size and the DARIS was too large, we adjusted the assumptions by increasing the anticipated intervention effect (by increasing relative risk reduction) to decrease the distance between the accrued information size and the DARIS (post hoc analyses). This was only conducted to make the TSA figures more easily interpretable. This reduces the apparent gap of required number of participants in further trials and does not affect our conclusions.

## Results

**Figure 1:**
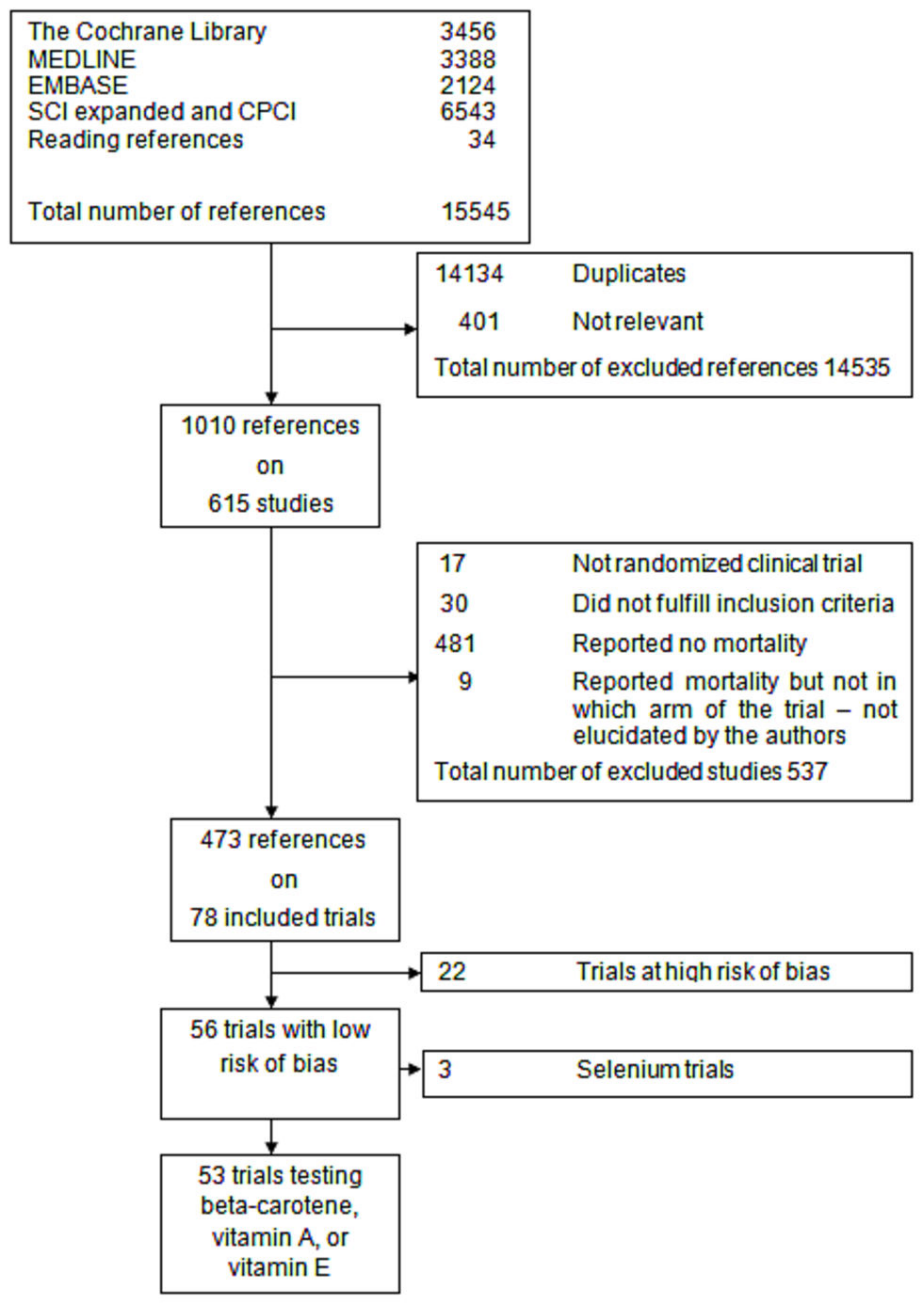
PRISMA flow diagram of identification of randomized clinical trials for inclusion.

We identified a total of 15,545 references of possible interest through searching the Cochrane Central Register of Controlled Trials (CENTRAL) in The Cochrane Library (n = 3456), MEDLINE (n = 3388), Embase (n = 2124), Science Citation Index Expanded (n = 6515), Conference Proceedings Citation Index-Science (n = 28), and reference lists (n = 34) (Figure 1) [8]. We excluded 14,134 duplicates and 401 clearly irrelevant references through reading the abstracts. Accordingly, 1010 references describing 615 trials were retrieved for further assessment. Of these, we excluded 47 studies because they were not randomized trials or did not fulfill our inclusion criteria. We included 568 randomized trials. The authors of 481 trials did not report mortality. The majority of these were small phase I or phase II trials, with a short duration of follow-up and without assessment of clinical outcomes. In nine trials [31–39] there were deaths reported, but the authors did not report in which group of the trial and did not respond to our requests for additional information [8].

In total, 78 randomized trials with 296,707 participants reported on mortality (Figure 1). Fifty-six trials (244,056 participants) were with low risk of bias [8]. Twenty-two trials (52,651 participants) were with high risk of bias and were excluded [40–61]. . [8]

Fifty-three randomized trials with low risk of bias assessed beta-carotene, vitamin A, and vitamin E singly, in any combination, or in combination with other vitamins and trace elements. They included 241,883 participants, aged 18 to 103 years, and 44.6% were women. Beta-carotene was assessed in 26 trials (173,006 participants), vitamin A in 12 trials (41,144 participants), and vitamin E in 46 trials (171,244 participants) (Table 1) [62–114]. All antioxidant supplements were administered orally. Beta-carotene was tested in a daily dose of 1.2 to 72.0 mg (mean 21 mg; median 19.5 mg); vitamin A was tested in a daily dose of 400 to 7500 µg (mean 2056 µg; median 800 µg); and vitamin E was tested in a daily dose of 10 to 5000 mg (mean 470 mg; median 350 mg). Two beta-carotene trials [63,79], two vitamin A trials [75,98], and two vitamin E trials [77,93] used doses above the TUIL. The antioxidant supplements were administered daily or on alternate days for 1 day to 12 years; weighted mean 6.9 years. The weighted mean duration of follow-up was 8.5 years (range 2.8 months to 14.1 years) (Table 1; Table 2).

**Table 1:** Characteristics of 53 Included Randomized Clinical Trials with Low Risk of Bias.

Trial	Design	No. of Participants	Women %	Mean Age	Suppl. (Y)	Follow-up (Y)	BC, mg	Vit A µg	Vit C mg	Vit E mg	Selenium µg
AREDS [62] 2001	2x2	4757	56	68	6.3	6.3	15		500	400	
ARMDS [63] 1996	Parallel	71	7	72	1.5	1.5	72		750	165	50
Allsup et al [64], 2004	Parallel	164	63	83	0.15	0.5		800	120	60	60
Avenell et al [65], 2005	Parallel	910	47	72	1	1		800	60	10	
Boaz et al [66], 2000	Parallel	196	31	65	1.42	1.42				537	
Brown et al [67], 2001	2x2	160	13	53	3	3	25		1000	537	100
Chylack et al [68], 2002	Parallel	297	59	68	3	3	18		750	600	
Collins et al [69], 2003	2x2	52	2	67	0.5	2.5				400	
Cook et al [70], 2007	2x2x2	8171	100	61	9.4	9.4	25		500	221	
Correa et al [71], 2000	2x2x2	976	54	51	6	6	30		2000		
Desnuelle et al [72], 2001	Parallel	288	45	64	1	1				1000	
Garbagnati et al [73], 2009	Parallel	72	35	65	1	1	19		240	290	
Girodon et al [74], 1999	2x2	725	74	84	2	2	6		120	15	100
Goodman et al [75], 2004	Parallel	18314	34	58	4	10	30	7500			
Graat et al [76], 2002	2x2	652	50	N/A	1	1	1.2	600	60	210	25
Graf et al [77], 2005	Parallel	160	35	58	1.5	1.5				5000	
Green et al [78], 1999	2x2	1621	56	49	4.5	4.5	30				
Greenberg et al [79], 1990	Parallel	1805	30	N/A	5	5	50				
Greenberg et al [80], 1994	2x2	864	21	61	4	4	25		1000	400	
Grieger et al [81], 2009	Parallel	115	65	NA	0.5	0.5	3		75	12,2	
Hennekens et al [82], 1996	2x2	22071	0	53	12	12.9	25				
Hercberg et al [83], 2010	Parallel	13017	61	49	7.54	7.54	6		120	30	100
Hodis et al [84], 2002	Parallel	353	52	56	3	3				364	
Jacobson et al [85], 2000	Parallel	112	42	42	0.5	0.5	12		500	268	
Lee et al [86], 2005	2x2	39876	100	55	10.1	10.1	25			221	
Li et al [87], 1993	Parallel	3318	56	54	6	6	15	3000	180	54	50
Lippman et al [88], 2008	2x2	35533	0	62	5.5	5.5				400	200
Liu et al [89], 2007	Parallel	763	68	85	1.6	1.6	16	400	80	44	20
Lonn et al [90], 2005	2x2	9541	27	66	4.5	7				294	
Magliano et al [91], 2006	Parallel	409	55	63	4	4				336	
Manuel-y-Keenoy et al [92], 2004	Parallel	24	14	51	0.5	4.5				503	
Marras et al [93], 2005	2x2	800	34	61	2.6	13				2000	
McNeil et al [94], 2004	Parallel	1193	56	66	4	4				500	
Meydani et al [95], 2004	Parallel	617	73	84	1	1				182	100
Mezey et al [96], 2004	Parallel	51	33	48	0.25	1				1000	
Milman et al [97], 2008	Parallel	1434	52	69	1.5	1.5				268	
Moon et al [98], 1997	Parallel	2297	30	63	3.8	3.8		7500			
Mooney et al [99], 2005	Parallel	284	45	37	1.25	1.25			500	364	
HPS [100] 2002	2x2	20536	25	N/A	5	5	20		250	600	
Murphy et al [101], 1992	Parallel	109	N/A	N/A	0.003	0.25		667			
Pike et al [102], 1995	Parallel	47	72	69	1	1		800	90	45	
Plummer et al [103], 2007	Parallel	1980	53	NA	3	3	18		750	600	
Prince et al [104], 2003	Cross-over	61	92	58	0.25	0.25	3		150	50	75
Richer et al [105], 2004	Parallel	61	4	75	1	1	9	750	1500	413	200
Salonen et al [106], 2003	2x2	520	51	N/A	6	6			250	182	
Sesso et al [107], 2008	2x2x2	14641	0	64	8	8	25	1050	560	182	20
Stephens et al [108], 1996	Parallel	2002	16	62	1.4	1.4				403	
Tam et al [109], 2005	Parallel	39	100	46	0.23	2.67			500	661	
Virtamo et al [110], 2003	2x2	29133	0	57	6.1	14.1	20			50	
Waters et al [111], 2002	2x2	423	100	65	3	3			1000	800	
White et al [112], 2002	Parallel	100	42	63	0.23	0.23			1000	200	
Witte et al [113], 2005	Parallel	32	N/A	N/A	0.75	0.75		800	500	400	50
Wluka et al [114], 2002	Parallel	136	45	64	2	2				368	

Abbreviations: N/A, Not Available; Suppl, Duration of Supplementation; Y, year; BC, Beta-carotene; Vit, Vitamin.

**Table 2:** Recommended dietary allowances, tolerable upper intake levels, and experimental doses used of antioxidant supplement.

**Antioxidant supplements**	RDA**			TUIL***	**Experimental doses**	**Median doses**
	**Men**	**Women**				
**Beta-carotene**	9.6 mg*			36 mg*	1.2 to 50 mg	19.5 mg
**Vitamin A**	900 µg		700 µg	3000 µg	400 to 60000 µg	800 µg
**Vitamin E**	15 mg		15 mg	1000 mg****	10 to 5000 mg	350 mg

^*^Calculated based on retinol equivalents.

^**^ **RDA The recommended dietary allowance** is the average daily dietary intake level that is sufficient to meet the nutrient requirement of nearly all (97 to 98 per cent) healthy individuals in a particular life stage and gender group [20,21].

^***^TUIL Tolerable upper intake level is the highest level of nutrient intake that is likely to pose no risk of adverse health effects for almost all individuals [22].

^****^The European Commission Scientific Committee on Food published its opinion on the tolerable upper intake level of vitamin E. The TUIL was established as 270 mg for adults, rounded to 300 mg [23].

Univariate and multivariate meta-regression analyses revealed that only the daily dose of vitamin A was significantly associated with the estimated intervention effect on mortality (univariate RR 1.00002, 95% CI 1.000004 to 1.00004, P = 0.017; multivariate RR 1.00002, 95% CI 1.000001 to 1.00004, P = 0.039) (Table 3). None of the other covariates (daily dose of beta-carotene; daily dose of vitamin E; primary or secondary prevention, single or combined administration) were significantly associated with the estimated intervention effect on mortality (Table 3).

**Table 3:** Meta-regression analyses on trials with low risk of bias.

**Covariates**	**Trials (N)**	**Univariate**				**Multivaraite**			
		**RR**	**95% CI**		**P**	**RR**	**95% CI**		**P**
**Dose of beta-carotene**	26	1.005	0.999	1.01	0.12	1.005	0.998	1.012	0.15
Type of prevention*		1.032	0.933	1.142	0.54	1.021	0.923	1.130	0.68
**Administered singly or combined**		1.003	0.907	1.109	0.95	0.990	0.900	1.089	0.83
**Dose of vitamin A**	12	1.00002	1.000004	1.00004	0.017	1.00002	1.000001	1.00004	0.039
Type of prevention*		1.111	0.880	1.403	0.38	1.156	0.910	1.470	0.24
**Administered singly or combined**		1.105	0.733	1.665	0.63	1.161	0.748	1.803	0.51
**Dose of vitamin E**	46	1.00002	0.999	1.0001	0.68	1.00002	0.999	1.0001	0.70
Type of prevention*		0.989	0.924	1.059	0.76	0.982	0.905	1.067	0.67
**Administered singly or combined**		1.028	0.960	1.100	0.44	1.039	0.964	1.120	0.31

Abbreviations: RR-relative risk; CI-confidence interval.

^*^Either primary prevention or secondary prevention.

### Meta-analyses and trial sequential analyses

#### Beta-carotene trials

Beta-carotene used singly versus placebo (7 trials, 43,019 participants) significantly increased mortality (RR 1.06, 95% CI 1.02 to 1.10, I^2^ = 0%) (Table 4).

**Table 4:** Type of antioxidant supplement, dose, all-cause mortality, accrued information size (AIS), and diversity-adjusted required information size (DARIS).

**Antioxidants**	Dose range and divided according to RDA*	**Trials N**	**All-cause mortality (RR, 95% CI)**	**AIS**	**DARIS**
**Beta-carotene singly**	25-50 mg	7	1.06 [1.02, 1.10]	43,019	110,505
**Beta-carotene singly or combined**	1.2-50 mg	26	1.05 [1.01, 1.09]	173,006	261,708
	≤ 9.6 mg*	6	0.90 [0.69, 1.17]	14,285	267,631
	> 9.6 mg*	20	1.06 [1.02, 1.09]	158,721	190,906
**Vitamin A singly**	667-7500 µg	2	1.18 [0.83, 1.68]	1323	110,505
**Vitamin A singly or combined**	400-60000 µg	12	1.07 [0.97, 1.18]	41,144	394,010
	≤ 800 µg*	8	1.05 [0.65, 1.69]	1415	456,748
	> 800 µg*	4	1.08 [0.98, 1.19]	38,570	415,996
**Vitamin E singly**	50-5000 mg	20	1.02 [0.98, 1.05]	58,904	110,505
**Vitamin E singly or combined**	10-5000 mg	46	1.03 [1.00, 1.05]	70,836	110,505
	≤ 15 mg*	2	1.32 [0.51, 3.46]	563	119,364
	> 15 mg*	44	1.03 [1.00, 1.05]	170,219	110,505

Abbreviations: RDA - recommended dietary allowance; CI – confidence interval; N – number of trials; n – number of participants; AIS – accrued information size; DARIS – diversity-adjusted required information size; n/a – not available

^*^calculated based on retinol equivalents

Beta-carotene used singly or in combination with other antioxidants versus placebo (26 trials, 173,006 participants) significantly increased mortality (RR 1.05, 95% CI 1.01 to 1.09, I^2^ = 21%) (Table 4).

**Figure 2:**
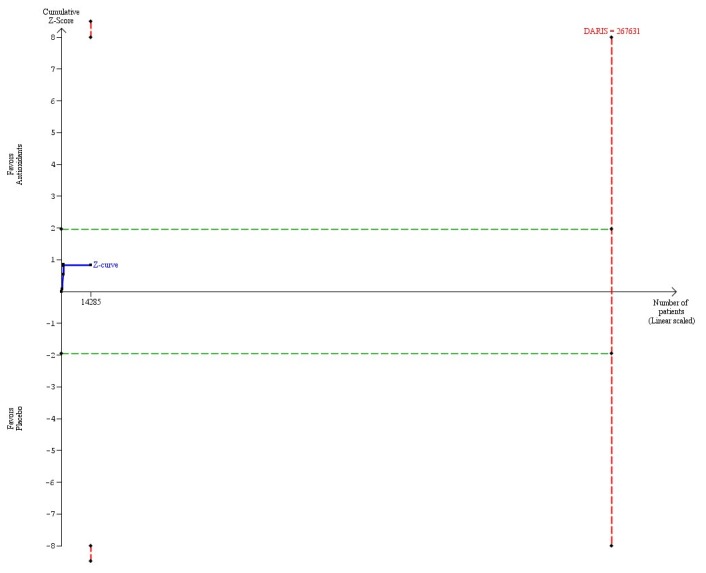
Trial sequential analysis of 6 trials assessing beta-carotene in a dose at or below 9.6 mg daily versus placebo. The diversity-adjusted required information size (DARIS = 267,631 participants) was based on a proportion of deaths of 10% in the placebo group; a relative risk reduction of 5% in the beta-carotene group; an alpha of 5%; a beta of 20%; and a diversity of 59%. The blue line represents the cumulative Z-score of the meta-analysis. The green lines represent the conventional statistical boundaries. The red lines represent the truncated trial sequential monitoring boundaries.

Beta-carotene used singly or in combination with other antioxidants in a dose at or below 9.6 mg (considered to be the RDA) versus placebo (6 trials, 14,285 participants) had no significant effect on mortality (RR 0.90, 95% CI 0.69 to 1.17, I^2^ = 23%) (Table 4). There was diversity (D^2^ = 59%). The DARIS was 267,631 participants (Table 4). Accordingly, only 5.3% of the DARIS were randomized. The trial sequential analysis of the six trials revealed that the cumulative Z-curve (blue line) did neither cross the conventional boundaries for significance (green lines) nor the trial sequential monitoring boundaries for harm or benefit (inward sloping red lines) (Figure 2). Neither did the cumulative Z-curve reach the trial sequential monitoring boundaries for futility (not even drawn by the program due to the large difference between the accrued information size and the DARIS).

**Figure 3:**
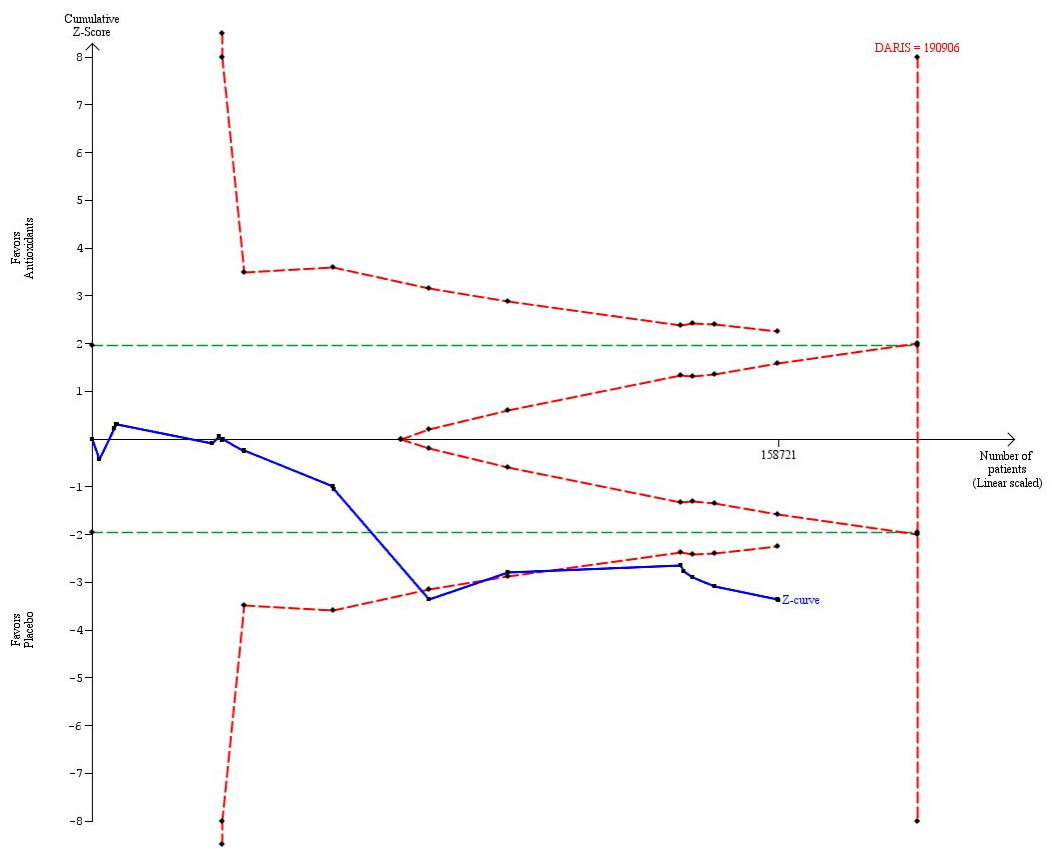
Trial sequential analysis of 20 trials assessing beta-carotene in a dose above 9.6 mg daily versus placebo. The diversity-adjusted required information size (DARIS = 190,906 participants) was based on a proportion of deaths of 10% in the placebo group; a relative risk reduction of 5% in the beta-carotene group; an alpha of 5%; a beta of 20%; and a diversity of 42%. The blue line represents the cumulative Z-score of the meta-analysis. The green lines represent the conventional statistical boundaries. The red inward sloping lines represent the trial sequential monitoring boundaries. The red outward sloping lines represents the area of futility.

Beta-carotene in a dose above 9.6 mg (considered to be the RDA) versus placebo (20 trials, 158,721 participants) significantly increased mortality (RR 1.06, 95% CI 1.02 to 1.09, I^2^ = 13%) (Table 4). There was diversity (D^2^ = 42%). The DARIS was 190,906 participants (Table 4; Figure 3). The trial sequential analysis revealed that the cumulative Z-curve (blue line) crossed both the conventional significant boundary for harm in 2003 during the 13^th^ trial (green line) and the trial sequential monitoring boundary for harm (inward sloping red line) also in 2003 during the 13^th^ trial (Figure 3).

The difference between the estimate of the effect of beta-carotene on mortality in trials using a dose of beta-carotene within the RDA and trials using a dose of beta-carotene above the RDA was not statistically significant (Chi^2^ = 1.48, P = 0.22).

#### Vitamin A trials

Vitamin A used singly versus placebo (2 trials, 2406 participants) had no significant effect on mortality (RR 1.18, 95% CI 0.83 to 1.68, I^2^ = 0%) (Table 4).

Vitamin A used singly or in combination with other antioxidants versus placebo (12 trials, 41,144 participants) had no significant effect on mortality (RR 1.07, 95% CI 0.97 to 1.18, I^2^ = 27%) (Table 4).

**Figure 4:**
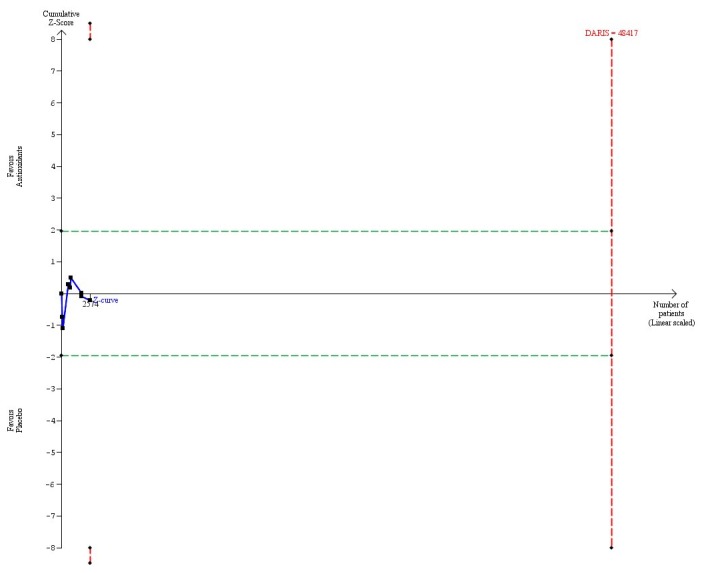
Trial sequential analysis of 8 trials assessing vitamin A in a dose at or below RDA (≤ 800 µg) daily versus placebo. The diversity-adjusted required information size (DARIS = 48,417 participants) was based on a proportion of deaths of 10% in the placebo group; a relative risk reduction of 15% in the vitamin A group; an alpha of 5%; a beta of 20%; and a diversity of 76%. The blue line represents the cumulative Z-score of the meta-analysis. The green lines represent the conventional statistical boundaries. The red lines represent the truncated trial sequential monitoring boundaries. Had we used a relative risk reduction of 5% as planned, the DARIS would have been 456,748 participants and the program could not have drawn the trial sequential analysis due to the fact that the number of randomized patients out of the DARIS is too small. This is why, we post hoc decided to construct the trial sequential analysis with a larger relative risk reduction.

Vitamin A in a dose at or below the RDA (≤ 800 µg) versus placebo (8 trials, 2574 participants) had no significant effect on mortality (RR 1.05, 95% CI 0.65 to 1.69, I^2^ = 15%) (Table 4). There was diversity (D^2^ = 76%). The DARIS was 48,417 participants based upon a relative risk reduction of 15% (post hoc estimation) and 456,748 participants based upon a priory relative risk reduction of 5% (Table 4; Figure 4). Accordingly, only 5.6% of the latter number of participants were randomized. The trial sequential analysis showed that the cumulative Z-curve (blue line) neither crossed the conventional boundaries for significance (green lines) nor the trial sequential monitoring boundaries for harm or benefit (inward sloping red lines). Neither did the cumulative Z-curve reach the trial sequential monitoring boundaries for futility (not even drawn by the program due to the large difference between the accrued information size and the DARIS even in the post hoc analysis) (Figure 4).

**Figure 5:**
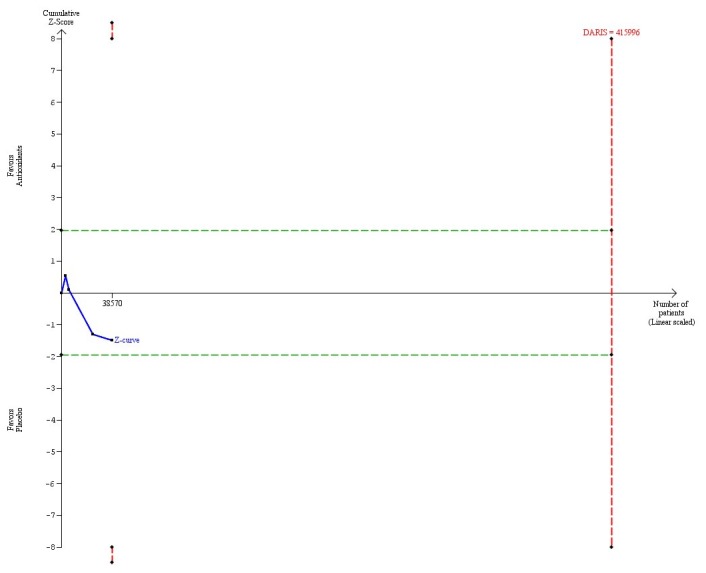
Trial sequential analysis of 4 trials assessing vitamin A in a dose above the RDA (> 800 µg) daily versus placebo. The diversity-adjusted required information size (DARIS = 415,996 participants) was based on a proportion of deaths of 10% in the placebo group; a relative risk reduction of 5% in the vitamin A group; an alpha of 5%; a beta of 20%; and a diversity of 73%. The blue line represents the cumulative Z-score of the meta-analysis. The green lines represent the conventional statistical boundaries. The red lines represent the truncated trial sequential monitoring boundaries.

Vitamin A in doses above the RDA (> 800 µg) versus placebo (4 trials, 38,570 participants) had no significant effect on mortality (RR 1.08, 95% CI 0.98 to 1.19, I^2^ = 53%) (Table 4). There was diversity (D^2^ = 73%). The DARIS was 415,996 participants (Table 4; Figure 5). Accordingly, only 9.2% of the DARIS were randomized. The trial sequential analysis showed that the cumulative Z-curve (blue line) neither crossed the conventional boundaries for significance (green lines) nor the trial sequential monitoring boundaries for harm or benefit (inward sloping red lines). Neither did the cumulative Z-curve reach the trial sequential monitoring boundaries for futility (not even drawn by the program due to the large difference between the accrued information size and the DARIS) (Figure 5).

The difference between the estimate of the effect of vitamin A on mortality in trials using a dose of vitamin A within the RDA and trials using a dose of vitamin A above the RDA was not significant (Chi^2^ = 0.01, P = 0.92).

#### Vitamin E trials

Vitamin E used singly versus placebo (20 trials, 58,904 participants) had no significant effect on mortality (RR 1.02, 95% CI 0.98 to 1.05, I^2^ = 0%) (Table 4).

Vitamin E used singly or in combination with other antioxidants versus placebo (46 trials, 171,244 participants) significantly increased mortality (RR 1.03, 95% CI 1.00 to 1.05, I^2^ = 0%) (Table 4).

**Figure 6:**
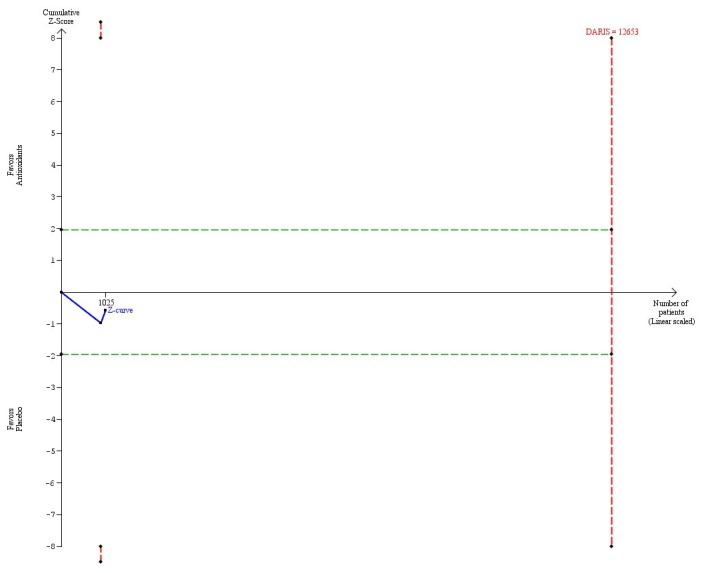
Trial sequential analysis of 2 trials assessing vitamin E in a dose at or below RDA (≤ 15 mg) daily versus placebo. The diversity-adjusted required information size (DARIS = 12,563 participants) was based on a proportion of deaths of 10% in the placebo group; a relative risk reduction of 15% in the vitamin E group; an alpha of 5%; a beta of 20%; and a diversity of 7%. The blue line represents the cumulative Z-score of the meta-analysis. The green lines represent the conventional statistical boundaries. The red lines represent the truncated trial sequential monitoring boundaries. Had we used a relative risk reduction of 5% as planned, the DARIS would have been 119,364 participants and the program could not have drawn the trial sequential analysis due to the fact that the number of randomized patients out of the DARIS is too small. This is why, we post hoc decided to construct the trial sequential analysis with a larger relative risk reduction.

Vitamin E in a dose at or below the RDA (≤ 15 mg) versus placebo (2 trials, 1025 participants) had no significant effect on mortality (RR 1.32, 95% CI 0.51 to 3.46, I^2^ = 7%) (Table 4). There was diversity (D^2^ = 7%). The DARIS was 119,364 participants (Table 4; Figure 6). Accordingly, only 0.9% of the DARIS were randomized. The trial sequential analysis showed that the cumulative Z-curve (blue line) neither crossed the conventional significant boundaries (green lines) nor the trial sequential monitoring boundaries for harm or benefit (inward sloping red lines). Neither did the cumulative Z-curve reach the trial sequential monitoring boundaries for futility (which were not even drawn by the program due to the large difference between the accrued information size and the DARIS even in the post hoc analysis) (Figure 6).

**Figure 7:**
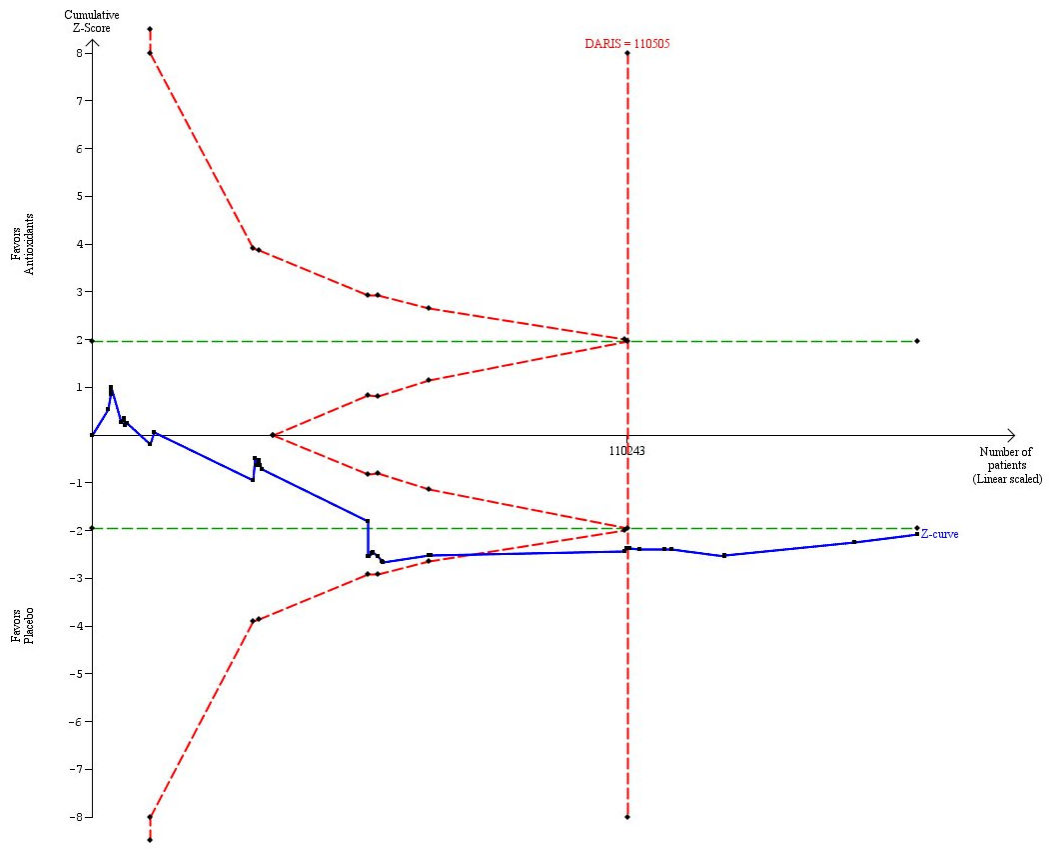
Trial sequential analysis of 44 trials assessing vitamin E in a dose above the RDA (> 15 mg) daily versus placebo. The diversity-adjusted required information size (DARIS = 110,505 participants) was based on a proportion of deaths of 10% in the placebo group; a relative risk reduction of 5% in the vitamin E group; an alpha of 5%; a beta of 20%; and a diversity of 0%. The blue line represents the cumulative Z-score of the meta-analysis. The green lines represent the conventional statistical boundaries. The red inward sloping lines represent the trial sequential monitoring boundaries. The red outward sloping lines represent the area of futility.

Vitamin E in a dose above the RDA (> 15 mg) versus placebo (44 trials, 170,219 participants) significantly increased mortality (RR 1.03, 95% CI 1.00 to 1.05, I^2^ = 0%) (Table 4). There was no diversity (D^2^ = 0%). The DARIS was 110,505 participants (Table 4; Figure 7). The trial sequential analysis showed that the cumulative Z-curve (blue line) crossed the conventional boundary for harm in 2003 during the 21^th^ trial and crossed the trial sequential monitoring boundary for harm (inward sloping red line) in 2005 during the 34^th^ trial (Figure 7).

The difference between the estimate of the effect of vitamin E on mortality in trials using a dose within the RDA and trials using a dose above the RDA was not significant (Chi^2^ = 0.27, P = 0.60).

## Discussion

Our study contains a number of new findings. Beta-carotene and vitamin E used singly or in combination with other antioxidants significantly increased mortality when administered in doses above the RDA. These findings were supported by trial sequential analyses, leaving out random errors. We could not draw any firm conclusion if doses of vitamin A above the RDA were harmful or not. The trial sequential analysis for vitamin A demonstrated that we do not have sufficient evidence, as the cumulative Z-curve did not cross the conventional boundaries, the trial sequential monitoring boundaries for benefit or harm, or the trial sequential monitoring boundaries for futility. Accordingly, the lack of benefit or harm could be a random error [13,28]. On the other hand, our meta-regression analysis showed that the dose of vitamin A was significantly associated with the estimated intervention effect on mortality. In support of this observation, when we meta-analyzed only the trials in which interaction or effect modification from the factorial design could be excluded, we observed a significant increase in mortality from vitamin A (RR 1.14, 95% CI 1.07 to 1.20; 10 trials, 26,015 participants), in accordance with our previous observations [7,8].

The effects of beta-carotene, vitamin A, and vitamin E on mortality seemed neutral when administered in doses within the RDAs. Again, the trial sequential analyses showed that we have insufficient information to show if the antioxidants within the RDAs provide benefits, harms, or have neutral effects. Absence of evidence is not evidence of absence of effect, be it beneficial or harmful [115]. The fact that we cannot find any significant differences between the intervention effects of meta-analyses of doses above the RDAs when compared to the intervention effects of meta-analyses of doses within the RDAs underlines further that we cannot assume lack of harm from smaller doses.

### Strengths

Our present study represents a comprehensive review of 53 randomized clinical trials with low risk of bias including almost a quarter million participants. This increases the precision and power of our analyses [15]. The outcome of all-cause mortality has been the primary outcome of our systematic review since publication of the first protocol in 2003 [8]. Previous meta-analyses of preventive trials of antioxidant supplements have included less information [116–118]. We only focused on trials with low risk of bias, leaving out trials that may overestimate benefits and underestimate harms [15–19]. Our meta-analyses had little trial heterogeneity. This further increases the trustworthiness of our findings. We also performed trial sequential analyses to estimate the risk of random errors in the cumulative meta-analyses and to prevent premature statements of superiority or inferiority of antioxidant supplements [9,11–13,27,28]. Hereby, we were able to assess the risks of random errors [9,11–13,27,28]. One should discuss how much evidence one would require when dealing with potential harm or harmful effects. On the one hand, harmful effects may occur due to random errors, and, therefore, sufficient information needs to be assessed to demonstrate harm beyond reasonable doubt. We have excluded such random error risks for doses above the RDA for beta-carotene and vitamin E. On the other hand, when the evidence is pointing towards harm, then we think, that ethical considerations should prevail and proof beyond any doubt should be avoided [119,120].

### Limitations

Our present study has several limitations. As with all systematic reviews, our findings and interpretations are limited by the quality and quantity of available evidence on the effects of specific supplements on mortality. A number of trials did not report mortality, although mortality is a very important part of serious adverse events and ought to be reported in all randomised trials [8]. We have extensively examined the influence of trials without any deaths on our results and found no noticeable effects [8]. The examined populations varied. The effects of supplements were assessed in general population or in patients with gastrointestinal, cardiovascular, neurological, skin, ocular, renal, endocrinological, rheumatoid, and undefined diseases in a stable phase [8]. We did not observe any differences in the effects of the beta-carotene, vitamin A, and vitamin E singly or in different combinations on mortality in these two groups [8]. These populations mostly came from countries without overt deficiencies of specific supplements. Accordingly, we are unable to assess how the antioxidant supplements affected mortality in populations with specific nutritional needs. Most trials assessed combinations of different supplements, which reflects the way supplements are marketed, sold, and taken by people [6,121,122]. As a result, we have compared antioxidants with different properties, given at different doses, and for different periods of time, either singly or combined. We are aware of the potential risks in assessing together the effects of different types of antioxidants with different mechanisms of action, biotransformation, and bioavailability. The methodological quality of some of the trials was assessed using the published reports, which may not reflect the actual design and risk of bias of the trials. Only some authors responded to our requests for further information [8]. Furthermore, our review includes several trials in which we cannot exclude interaction or effect modification by other interventions examined in these trials [8]. However, when we removed trials with potential interaction or effect modification then a statistically significant effect of vitamin A on all-cause mortality was revealed [8].

Most trials investigated the effects of supplements administered at higher doses than those commonly found in a balanced diet using doses well above the RDA. Some trials assessed doses even above the upper tolerable intake levels (Table 2) [20,21]. Too low intake of certain vitamins and minerals may lead to specific deficiency syndromes. However, too high intakes are associated with adverse health effects [123].

Our results extend the evidence in previous reviews suggesting that beta-carotene, vitamin A, and vitamin E may be harmful [117,118,124]. There are several possible explanations for the negative effect of antioxidant supplements on mortality [125,126]. Although oxidative stress has a hypothesized role in the pathogenesis of many chronic diseases, it may be the consequence of pathologic conditions [127,128]. By eliminating free radicals from our organism, we interfere with some essential defensive mechanisms like apoptosis, phagocytosis, and detoxification [125,126,129]. Antioxidant supplements are synthetic and not subjected to the same rigorous toxicity studies like other pharmaceutical agents [130]. Better understanding of mechanisms of action, biotransformation, bioavailability, safety, and appropriate dosage of antioxidants in relation to potential disease prevention is needed. [131] A balanced diet can provide sufficient and healthy amounts of vitamins [3,4]. Today, more than one half of adults in high-income countries ingest dietary supplements [6,132], and most frequently in the form of multivitamins with or without minerals [122]. When combined with dietary intake, the total intake of vitamin A and vitamin E of antioxidant supplement users in the United States exceeds 100% of the estimated average requirement, with the intake of vitamin E exceeding 700% of the estimated average requirement [122,133,134]. Bailey et al. recently reported that 5% of adults in the United States older than 50 years exceed the vitamin A tolerable upper intake level [132]. Therefore, there is a clear need for a risk assessment of antioxidant supplements because the total daily intake of certain vitamins and minerals may reach critical levels. [135] One should consider the U-shaped relation between vitamin status and mortality risk [123,136,137]. Antioxidant vitamins may have adverse health effects at both too low and too high intakes. Lack of a vitamin due to inadequate intake, malabsorption, or increased excretion may lead to deficiency. In this situation, it seems rational to use vitamin supplementation. However, excessive supplementation in people already well saturated with antioxidants through their diet seem to provoke adverse health effects [123,138,139].

Our results are consistent with the 2010 Dietary Guidelines for Americans, as well as conclusions of a National Institutes of Health-sponsored State-of-the-Science Conference that there is no evidence to support the use of multivitamin/mineral supplements in the primary prevention of chronic diseases [140,141]. There is no convincing evidence showing benefits of antioxidant supplements on disease prevention; cancer occurrence; cardiovascular outcomes; or quality of life.

## Conclusions

The current evidence calls for a shift in attitude towards antioxidant supplements, with beta-carotene, vitamin A, and vitamin E in specific. The current evidence on the effects of these antioxidants on all-cause mortality, disease occurrence, and quality of life does not support the use of these antioxidant supplements in a generally well-nourished population [142–144]. Beta-carotene, vitamin A, and vitamin E in lower doses may have neutral or beneficial effects on mortality, but according to our accumulated evidence we cannot exclude harmful effects either. We simply lack such knowledge.
